# Tirzepatide Improves Early Dumping Syndrome and Glucose Nadir in Postbariatric Hypoglycemia After Sleeve Gastrectomy

**DOI:** 10.1210/jcemcr/luae194

**Published:** 2024-10-23

**Authors:** Ethan Stortz, Helen Lawler

**Affiliations:** Division of Endocrinology, Metabolism, and Diabetes, University of Colorado School of Medicine, Aurora, CO 80045, USA; Division of Endocrinology, Metabolism, and Diabetes, University of Colorado School of Medicine, Aurora, CO 80045, USA

**Keywords:** postbariatric hypoglycemia, early dumping syndrome, bariatric surgery, GLP-1, GIP, tirzepatide

## Abstract

Early dumping syndrome (DS) and postbariatric hypoglycemia (PBH) are challenging conditions with limited treatment options. A 46-year-old woman with prediabetes, obesity, and sleeve gastrectomy presented with digestive symptoms suggestive of DS and postprandial hypoglycemia consistent with PBH. She started tirzepatide 2.5 mg weekly, which decreased postprandial blood glucose peaks, increased postprandial blood glucose nadirs, and improved overall time in range on continuous glucose monitoring (CGM). Her postprandial bloating and diarrhea resolved. To our knowledge, there have been no reported cases of DS or PBH treated with dual-incretin agonists. While glucagon-like peptide-1 (GLP-1) agonists have not been widely attempted in DS and have shown mixed benefit for PBH, combination GLP-1 and gastric inhibitory peptide agonism may represent a novel treatment both for PBH and DS, providing greater improvement in glycemic variation as well as better DS control than GLP-1 agonism alone.

## Introduction

Early dumping syndrome (DS) and postbariatric hypoglycemia (PBH) are challenging complications of Roux-en-Y gastric bypass (GB), sleeve gastrectomy (SG), and other upper gastrointestinal surgeries, such as Nissen fundoplication. Commonly employed treatments vary in efficacy. We describe a case in which tirzepatide successfully improved symptoms of DS and PBH.

DS refers to a constellation of gastrointestinal and vasomotor symptoms that occur 15 to 30 minutes after eating. These symptoms include nausea, bloating, cramping, abdominal pain, or diarrhea along with flushing, lightheadedness, hypotension, tachycardia, or syncope [1]. Symptoms usually start shortly after surgery and are prevalent in up to 40% of patients after any bariatric procedure [2]. Reduced gastric volume leads to rapid delivery of concentrated, hyperosmolar gastric contents to the small intestine. This causes rapid fluid absorption into the intestines from the intravascular space, decreasing circulating blood volume and increasing digestive peptide hormone and vasoactive hormone release.

One study suggested that elevated glucagon-like peptide 1 (GLP-1) and peptide YY levels after a mixed meal correlate with DS symptoms [3]. GLP-1 may enhance sympathetic output based on its activity in the central nervous system, which in turn could lead to symptoms seen in DS [4]. Treatment of DS consists of dietary modifications including eating small, frequent meals, chewing thoroughly, and avoiding simple carbohydrates. Liquids should be avoided 30 minutes before and 60 minutes after food consumption [1]. Case reports suggest octreotide is an effective treatment option for those failing dietary modification alone [5]. While some clinicians speculate that GLP-1 agonist medications may also provide benefit, evidence is lacking.

PBH, formerly known as “late dumping syndrome,” is becoming a well-known complication of bariatric surgery. Symptom onset usually occurs 1 year after surgery where other etiologies must be considered if the onset is less than 6 months post surgery. Patients experience symptomatic hypoglycemia 1 to 3 hours after eating [6]. Unlike DS, which does not cause hypoglycemia and usually dissipates within the first 6 months after surgery, PBH does not resolve. Symptoms of hypoglycemia occur in 33% of patients after SG and 38% of patients after Roux-en-Y GB, with 12% of patients experiencing severe hypoglycemic symptoms [6, 7]. It is not completely understood why postprandial dysglycemia transpires. The pathophysiology is likely multifactorial, but exaggerated GLP-1 release in response to intestinal glucose delivery causing hyperinsulinemia is one factor thought to play an important role [8].

**Figure 1.**
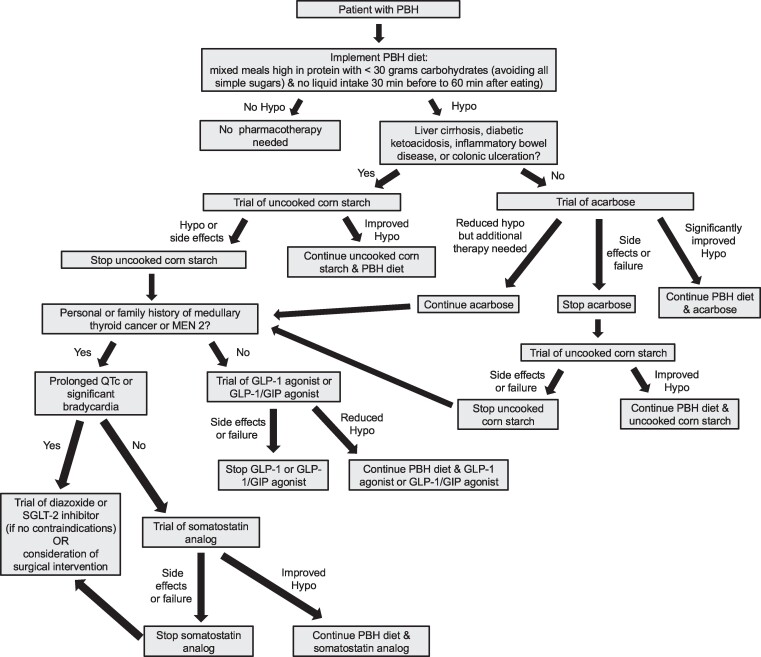
How we approach pharmacotherapy selection for treatment of postbariatric hypoglycemia (PBH). Abbreviations: hypo, hypoglycemia; GIP, gastric inhibitory peptide; GLP-1, glucagon-like peptide-1; SGLT-2, sodium-glucose cotransporter-2.

First-line treatment of PBH is dietary modification with avoidance of all simple sugars and consumption of mixed meals high in protein and low in complex carbohydrates (<30 g per meal). Continuous glucose monitoring (CGM) is a useful tool for patients with PBH as it has been associated with reduced glycemic variability and allows for earlier intervention to prevent hypoglycemia [9]. If dietary modification is not successful in minimizing symptoms, medications such as acarbose, GLP-1 agonists, sodium-glucose cotransporter-1/2 inhibitors, calcium channel blockers, diazoxide, or somatostatin analogues have been used with mixed results [10]. The mechanisms of action of these drugs are outlined in Table 1, and guidance for their use is presented in Fig. 1.

**Table 1.** Mechanism of action of pharmacotherapy used for postbariatric hypoglycemia

Pharmacotherapy	Mechanism of action in PBH
α-Glucosidase inhibitor (ie, acarbose)	Acts at the intestinal brush border to prevent polysaccharide hydrolysis to simple sugars which delays/reduces the absorption of dietary carbohydrates, attenuating plasma glucose and subsequent insulin rise to prevent postprandial hypoglycemia
Uncooked corn starch	Complex carbohydrate that slows digestion, limiting glucose peaks and subsequent hypoglycemia
GLP-1 agonist GLP-1/GIP agonist	Unknown mechanism of action in PBH
Somatostatin analogue	Inhibits postprandial hormone secretion of insulin, GLP-1, and other enteric hormones
Nondiuretic benzothiadiazine derivative (diazoxide)	Activates potassium channels of pancreatic beta cells leading to hyperpolarization, which subsequently inactivates calcium channel activity and reduces insulin secretion
SGLT-1 and SGLT-2 inhibitors	SGLT-1 inhibitors reduce intestinal glucose absorption, leading to lower glucose peaks. SGLT-2 inhibitors reduce reabsorption of sodium and glucose from the proximal tubules also resulting in lower postprandial hyperglycemia
Calcium channel blockers	Inhibits calcium channels of pancreatic β cells, which reduces insulin secretion

Abbreviations GIP, gastric inhibitory peptide; GLP-1, glucagon-like peptide-1; PBH, postbariatric hypoglycemia; SGLT-1, sodium-glucose cotransporter-1; SGLT-2, sodium-glucose cotransporter-2.

A systematic review of GLP-1 agonists for treatment of PBH showed that some patients experienced fewer hypoglycemic episodes while others had no change in symptoms or glucose parameters [11]. It is possible GLP-1 agonist medications may interrupt PBH pathophysiology primarily by acting as an effective “GLP-1 clamp” where exogenous GLP-1 at consistent plasma concentrations may reduce the amplified GLP-1 receptor activation and subsequent insulin release in response to intestinal glucose delivery [11]. Stable GLP-1 levels may also decrease GLP-1 agonist–mediated sympathetic changes in response to the delivery of hyperosmolar gastric contents to the small intestine [4]. The effect of gastric inhibitory peptide (GIP) in mediating PBH and early dumping is less clear. GIP concentrations have been shown to increase, remain stable, and/or decrease after GB, while they increase or remain stable after SG [12, 13].

## Case Presentation

**Figure 2.**
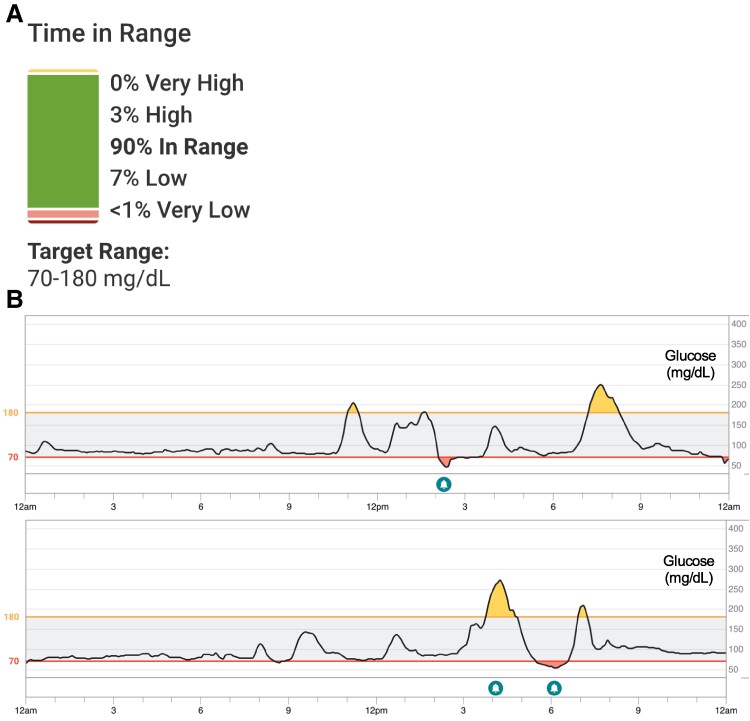
A, Continuous glucose monitor data prior to initiation of tirzepatide. Data covered a 7-day period. Average plasma glucose was 95 mg/dL (5.3 mmol/L) with coefficient of variation 33.3%. B, Sample continuous glucose data from 2 days showing postprandial hyperglycemia followed by subsequent hypoglycemia associated with postbariatric hypoglycemia (PBH) prior to the initiation of tirzepatide. Blood glucose values on Y-axis shown in mg/dL.

A 46-year-old woman with prediabetes, obesity, and SG presented with labile blood sugars, postprandial hypoglycemia, and intermittent severe bloating with diarrhea shortly after eating meals. She originally underwent a gastric banding procedure for weight loss 16 years ago when she weighed 253 lbs (115 kg, body mass index [BMI] 45). She lost approximately 80 lbs (36 kg), but her postoperative course was complicated by chronic vomiting. Because of these symptoms and weight regain to 211 lbs (96 kg), she underwent band removal and conversion to SG 2 years prior to presentation. While this resulted in resolution of her vomiting, she developed new symptoms of abdominal bloating and diarrhea occurring about 20 minutes post meal. Dietary modifications did not improve her symptoms. About 8 months post surgery, she noticed postprandial hunger, confusion, tremors, and fatigue. She was concerned that hypoglycemia was causing these symptoms, so she purchased a glucometer, which showed postprandial blood glucose peaks to 300 to 325 mg/dL (16.7-18.0 mmol/L) followed by blood glucose nadirs to 30 to 50 mg/dL (1.7-2.8 mmol/L). She acquired her own Dexcom G6 CGM a few weeks prior to her clinic visit, revealing postprandial hyperglycemic and hypoglycemic events (Fig. 2). Because she had acquired her own CGM and did not meet insurance requirements for coverage, she wore the device intermittently, often for week-long intervals, prohibiting assessment of consecutive 14- or 30-day ambulatory glucose profiles. She noticed ingestion of simple sugars caused peaks followed by plummets in her blood glucose. She attempted eating smaller, high-protein meals while avoiding simple sugars. These dietary changes resulted in only slight improvement and did not help her gastrointestinal symptoms. She also reported weight loss to 199 lbs (90 kg, BMI 35), which she attributed to her SG and dietary modifications. She denied alcohol use, other recreational substance use, and family history of hypoglycemia or neuroendocrine tumors. For her prediabetes, she took metformin 500 mg twice daily. Her only other prescription medication was bupropion for depression. Her over-the-counter supplements included calcium and a bariatric multivitamin.

## Diagnostic Assessment

Because the patient's history and presentation were consistent with PBH, further evaluation for other etiologies of hypoglycemia (eg, morning cortisol, insulin antibodies, 24-hour fast) was not pursued. She had a recent normal comprehensive metabolic panel and complete blood count. Relevant laboratory data from her initial visit is presented in Table 2. No imaging was indicated.

**Table 2.** Laboratory data at initial clinic visit

Variable	On initial evaluation	Reference range
Glycated hemoglobin	5.7% (39 mmol/mol)	4.0-5.6% (20-38 mmol/mol)
Triglycerides, nonfasting	214 mg/dL (2.42 mmol/L)	48-149 mg/dL (0.54-1.68 mmol/L)
LDL	128 mg/dL (3.3 mmol/L)	57-129 mg/dL (1.5-3.3 mmol/L)
HDL	44 mg/dL (1.1 mmol/L)	40-50 mg/dL (1.0-1.3 mmol/L)
TSH	1.71 μIU/L (1.71 mIU/L)	0.45-5.33 μIU/L (0.45-5.33 mIU/L)
Hemoglobin	12.5 g/dL (125 g/L)	12.1-16.3 g/dL (121-163 g/L)
MCV	84.7 µm^3^ (84.7 fL)	80-100 µm^3^ (80.0-100.0 fL)

Abbreviations: HLD, high-density lipoprotein; LDL, low-density lipoprotein; MCV, mean corpuscular volume; TSH, thyrotropin.

## Treatment

She was started on tirzepatide 2.5 mg daily. With documentation of a postprandial plasma glucose level greater than 200 mg/dL (11.1 mmol/L), she met criteria for diagnosis of diabetes and received insurance coverage of tirzepatide. A GLP-1/GIP agonist was chosen over GLP-1 agonist due to better efficacy with weight loss as her BMI was 35.

## Outcome and Follow-up

**Figure 3.**
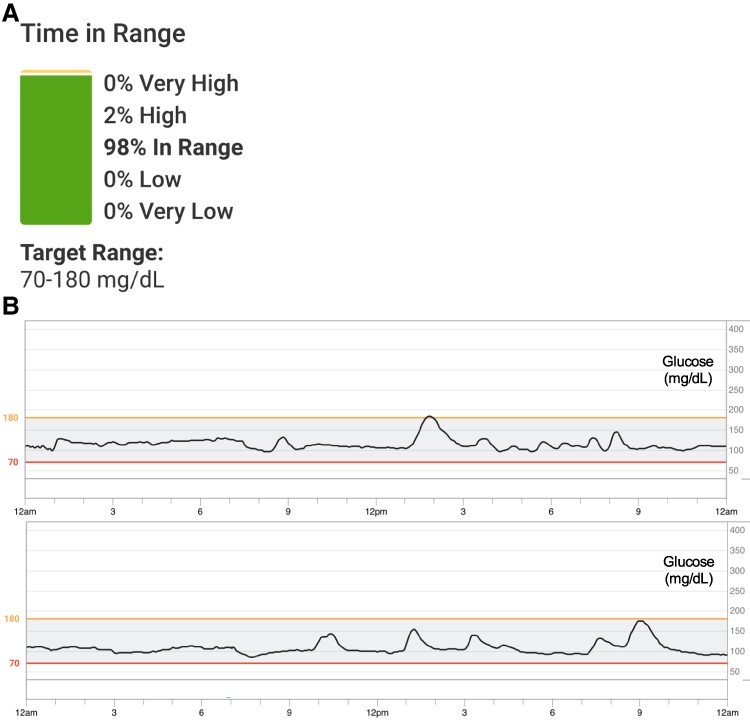
A, Continuous glucose monitor data during the fourth week of tirzepatide therapy. Data covered 6 days. Average plasma glucose was 110 mg/dL (6.1 mmol/L) with a coefficient of variation of 20.5%. B, Sample glucose data from 2 days showing improved hyperglycemia and hypoglycemia while on tirzepatide therapy. Blood glucose values on Y-axis shown in mg/dL.

After 4 weeks on tirzepatide 2.5 mg weekly, the patient experienced a reduction in postprandial blood glucose peak from 320 mg/dL (17.8 mmol/L) to 110 mg/dL (6.1 mmol/L) (normal peak usually < 140 mg/dL or 7.8 mmol/L) [14]. Her postprandial blood glucose nadir improved from 39 mg/dL (2.2 mmol/L) to 72 mg/dL (4.0 mmol/L) (normal >55 mg/dL or 3.1 mmol/L) [14]. Her time in range on CGM improved from 90-93% to 98% (Fig. 3). Her coefficient of variation was the lowest it had ever been at 20.5%, down from 33.3% prior to initiating tirzepatide. Subjectively, she reported fewer hypoglycemic symptoms. After 4 weeks of tirzepatide, her weight decreased to 191 lbs (87 kg, BMI 34) from 199 lbs (90 kg, BMI 35). Her glycated hemoglobin A_1c_ decreased from 5.7% (38 mmol/mol) to 5.4% (36 mmol/mol). Her DS symptoms significantly improved, with resolution of postprandial bloating and diarrhea.

Over the past 1.5 years, we have followed the patient in clinic where she has had no recurrence of DS and drastic improvement in PBH on tirzepatide therapy. Although tirzepatide 2.5 mg weekly controlled her symptoms, she opted to increase the dose to 5 mg weekly to assist with further weight loss. Her most recent weight is 160 lbs (73 kg, BMI 28) and her glycated hemoglobin A_1c_ is now 5.0% (31 mmol/mol). Interestingly, when she could not obtain tirzepatide for 6 weeks due to insurance coverage issues, she reported return of DS-related diarrhea and frequent episodes of PBH. These symptoms resolved with reinitiation of tirzepatide.

## Discussion

This patient had resolution of DS and improvement of PBH with tirzepatide administration, including lower glucose peaks and higher glucose nadirs. Incretin hormones play a substantial role in the pathophysiology of PBH and possibly DS. Meal-induced GLP-1 secretion is 10-fold higher in patients after GB compared to nonsurgical individuals. Patients who have undergone SG also have elevated GLP-1 levels, though not as high as those seen after GB [13, 15]. In patients without diabetes who underwent GB, β-cell sensitivity/responsiveness to exogenous incretin infusion following oral glucose ingestion was decreased compared to BMI-matched, nonsurgical controls [16]. A study looking at GLP-1 receptor (GLP-1R) and GIP receptor (GIPR) blockade in GB and SG patients showed that GLP-1R blockade reduced β-cell sensitivity and increased postprandial glucose responses, an effect that was not observed in nonsurgical controls [13]. This suggests an adaptive response occurs after bariatric surgery to help prevent hypoglycemia. However, this adaptation is aberrant in patients with PBH, and further study is needed to better understand why this occurs.

As previously mentioned, GLP-1 agonists may stabilize GLP-1 levels in patients with PBH, attenuating the hyperinsulinemic β-cell response and minimizing postprandial hypoglycemia. Interestingly, avexitide, a GLP-1 antagonist, reduced postprandial insulin secretion and subsequent hypoglycemia in PBH, but this is not yet approved by the USA Food and Drug Administration [10]. The fact that both GLP-1 agonists and antagonists have shown improvement in PBH-related hypoglycemia highlights the complexity of the condition's pathophysiology.

The role of GIP agonism in improvement of PBH is less clear. Reduction in glucagon levels in response to hypoglycemia have been documented in patients after GB, and those patients with PBH may have a higher degree of dysregulation [17]. It is possible that tirzepatide may have a different effect in patients who have undergone SG compared to those who have had GB. Because GIP production occurs mainly in the proximal intestine, which is bypassed in GB, and GLP-1 production occurs primarily in the distal small intestine, a “GIP clamp”-like effect may be more efficacious in patients after SG. In the aforementioned study using GLP-1R and GIPR blockade, GIP levels were lowest in patients after GB. GIPR blockade reduced β-cell sensitivity and increased postprandial glucose responses in SG and nonsurgical groups but had no effect in the GB group [13].

To our knowledge, this is the first case reporting the use of the dual-incretin agonist tirzepatide for treatment of PBH and DS. While case reports and small studies have not shown consistent benefit of GLP-1 agonists for PBH, tirzepatide may represent an efficacious option, especially for patients with PBH after SG. The addition of GIP may enhance glucagon secretion in response to meals, but the mechanism by which GIP may blunt serum glucose excursions and nadirs is not entirely understood. It is less clear how the addition of GIP to GLP-1 improves DS, but given how few pharmacotherapies exist for this condition, tirzepatide could provide a promising therapeutic option for these debilitating postsurgical conditions.

## Learning Points

- Early DS occurs shortly after bariatric surgery and consists of gastrointestinal and vasomotor symptoms that occur 15-30 minutes after eating and usually resolve within the first 6 months after surgery.
- The pathophysiology of DS stems from reduced gastric volume causing rapid delivery of hyperosmolar contents to the small intestine causing rapid fluid absorption, decreased circulating blood volume, and increased vasoactive hormone release.
- PBH, formerly known as “late dumping syndrome,” occurs at least 6 months after bariatric surgery where patients experience postprandial hypoglycemia causing neuroglycopenic symptoms. PBH can improve with dietary modification ± off-label medications but never spontaneously resolves.
- The pathophysiology of PBH is complex and multifactorial, but it is well known that incretin levels are involved, and patients experience postprandial hyperinsulinemic hypoglycemia.
- There are no USA Food and Drug Administration–approved medications to treat DS or PBH, yet this case report shows promise for tirzepatide as a potential treatment.

## Contributors

Both authors made individual contributions to authorship. E.S. and H.L. were involved in the diagnosis and management of the patient and manuscript submission. Both authors reviewed and approved the final draft.

## Data Availability

Original data generated and analyzed during this study are included in this published article.

## Abbreviations

BMI: body mass index
CGM: continuous glucose monitoring
DS: dumping syndrome
GB: gastric bypass
GIP: gastric inhibitory peptide
GIPR: gastric inhibitory peptide receptor
GLP-1: glucagon-like peptide-1
GLP-1R: glucagon-like peptide-1 receptor
PBH: postbariatric hypoglycemia
SG: sleeve gastrectomy
SGLT-1: sodium-glucose cotransporter-1
SGLT-2: sodium-glucose cotransporter-2
